# Cluster randomised controlled feasibility study of HENRY: a community-based intervention aimed at reducing obesity rates in preschool children

**DOI:** 10.1186/s40814-021-00798-z

**Published:** 2021-02-26

**Authors:** Maria Bryant, Michelle Collinson, Wendy Burton, Elizabeth Stamp, Holly Schofield, Bethan Copsey, Suzanne Hartley, Edward Webb, Amanda J. Farrin

**Affiliations:** 1 Department of Health Sciences, University of York, York, YO10 5DD UK; 2 Hull York Medical School, University of York, York, YO10 5DD UK; 3 Clinical Trials Research Unit, Leeds Institute of Clinical Trials Research, University of Leeds, Leeds, LS2 9JT UK; 4 National Centre of Sport and Exercise Medicine, Loughborough University, Epinal Way, Loughborough, Leicestershire, LE11 3TU UK; 5 Academic unit of Health Economics, Leeds Institute of Health Sciences, University of Leeds, Leeds, LS2 9JT UK

**Keywords:** Childhood obesity, Community, Prevention, Parent programme, Public health

## Abstract

**Background:**

Community-based obesity prevention interventions are often commissioned despite the limited evidence base. HENRY (Health, Exercise, Nutrition for the Really Young) is a programme delivered to parents of preschool children across the UK. Early evidence suggests that it may be effective, but a robust evaluation has not been conducted. We initiated a systematic evaluation of HENRY by studying the feasibility of conducting a multi-centre definitive trial to evaluate its effectiveness and cost-effectiveness to prevent obesity. Objectives were to assess the feasibility of recruiting local authorities, centres and parents; test processes and time required to train and certify intervention staff; explore HENRY commissioning processes; identify potential sources (and associated impact) of contamination; and consider the feasibility of trial procedures.

**Methods:**

We conducted a multi-centre, open labelled, two group, prospective, cluster randomised, controlled, feasibility study, with embedded process evaluation and pre-defined criteria for progression to definitive trial. We sought to recruit 120 parents from 12 children’s centres, across two UK local authority (government) areas. Within each local authority, we planned to randomise three centres to HENRY and three to ‘standard care’ control. Our plan was to collect data in family homes at baseline and 12 months, including parent and child height and weight, and parent-reported questionnaires on self-efficacy, feeding, eating habits, quality of life and resource use. Contamination, implementation and study acceptability were explored using parent interviews.

**Results:**

We recruited two local authorities and 12 children’s centres within eight months. One hundred and seventeen parents were recruited (average 3.9 parents per programme) and follow-up data were collected from 85% of participants. Process data from 20 parents and 24 members of staff indicate that both would benefit from more detail about their involvement as participants, but that methods were acceptable. Contamination was likely, though the impact of this on behaviour was unclear.

**Conclusion:**

Our findings indicate that a cluster RCT of HENRY to assess its effect on childhood obesity prevention is feasible. This study has allowed us to design a pragmatic definitive trial with minimal bias, taking account of lessons learnt from conducting evaluation research in public health settings.

**Trial registration:**

ClinicalTrials.gov Identifier NCT03333733 registered 6th November 2017.

**Supplementary Information:**

The online version contains supplementary material available at 10.1186/s40814-021-00798-z.

Key messages

- What uncertainties existed regarding the feasibility?

Trials within public health settings are needed to determine the effectiveness and cost-effectiveness of programmes that are widely funded by local government authorities. In our research of a childhood obesity prevention programme ‘HENRY’, key uncertainties related to the acceptability of the process, and our ability to recruit parents, children’s centres and local government authorities; the latter of which would need to agree to commission HENRY and allow nominated children’s centres to be randomly allocated to HENRY or control. Centres delivering such programmes are usually determined by population needs.

- What are the key feasibility findings?

The feasibility study demonstrated our ability to recruit local authorities, children’s centres and parents and that our data collection protocol was acceptable. We have demonstrated that it is possible to implement high-quality research in community based settings. Selection bias may have occurred through a targeted approach to parent enrolment to the intervention, even though it is designed to be available to all families. Staff appeared to present the highest risk of contamination, though the impact of this was uncertain.

- What are the implications of the feasibility findings for the design of the main study?

This research demonstrated that it was feasible to implement a robust design to test the effectiveness of a government disseminated obesity prevention programme for community settings, including specifying eligibility criteria to avoid selection bias and improving training to reduce missing height measurement data required to calculate BMI.

## Introduction

Childhood obesity rates have reached a concerning level and continue to rise; the incidence of children in the UK who are obese when starting school has reached 9.4% [1]. Thus, addressing the rising prevalence of childhood obesity is a health priority [1], particularly considering the government aim of halving childhood obesity by 2030 [2]. Childhood obesity can have severe implications on a child’s physical (e.g. prediabetes, high blood pressure) [3] and mental health [4] which can continue into adulthood [1, 5]. Adopting healthy behaviours in the early years of a child’s life ensures good development and growth [5]. Recently, the importance of obesity prevention during childhood has been highlighted as an effective strategy to avoid excess weight gain in later life [1, 6], particularly as obesity is difficult to reverse once established [7–9].

Though modest benefits have been observed [10], inconsistent findings have been reported for the effectiveness of pre-school obesity prevention programmes [7, 9] and many have not been rooted in behaviour change theory [11]. There is evidence that multicomponent interventions, particularly those engaging parents/carers are most effective [12]. One such programme is HENRY (Health, Exercise and Nutrition for the Really Young), a UK community-based pre-school obesity prevention programme that incorporates a number of behavioural techniques to improve lifestyle behaviours of parents and their pre-school aged children. The programme has collaborated with partners such as the NHS and local authorities, and works with over 1000 health and early years practitioners. Approximately 40 local authorities across the UK currently commission HENRY.

Although some research has been conducted into the effectiveness of the HENRY programme [13, 14] via routinely collected data [14, 15] and a cohort design [13], there has been no independent randomised controlled trial (RCT) evaluation. The MRC framework for complex interventions suggests a feasibility study should be conducted [16] to identify potential challenges or difficulties to be addressed before a definitive trial is conducted [16], and to refine the trial design and the intervention under evaluation [17]. This is particularly relevant here, given the complexities of the behaviour change intervention and the evaluation setting. We conducted an independent study with the primary aim of determining the feasibility of undertaking a definitive trial to evaluate the clinical and cost-effectiveness of the HENRY programme in preventing childhood obesity.

## Methods

### Design

A UK multi-centre, open labelled, two group, prospective, cluster randomised, controlled feasibility study, with a process evaluation conducted between July 2017 and November 2019. The research was approved by the University of Leeds School of Medicine Research Ethics Committee (MREC: 16-107) and registered on the clinical trial website (clinicaltrials.gov) #NCT03333733.

The study methods have been reported in detail elsewhere [18] and are presented more briefly below. Following study commencement, three key protocol amendments were made: (1) follow-up was reduced from 12 to 11 months due to an unexpected delay in the ethical approval process (approved by the Trial Steering Committee); (2) additional consent was requested at follow-up to access HENRY programme attendance data; and (3) telephone interviews were conducted with parents in the process evaluation to replace focus groups, which were not well attended.

### Objectives

Primary objectives were:

1. To determine whether it was feasible to recruit local authorities/service providers, childcare centres and parents, based on ability to meet predefined progression criteria
2. To assess the time required to train and certify staff to competently deliver HENRY programmes
3. To explore local area preferences for HENRY commissioning, provision and delivery via screening questionnaires and qualitative data collection in areas currently delivering HENRY
4. To explore potential sources and risks of contamination, including the degree to which parents used multiple centres, the level of contamination resulting from social networks (control and HENRY parents sharing knowledge) and the possibility of HENRY trained facilitators sharing knowledge with control centres.

Secondary objectives of the feasibility study were:

(5) To examine the acceptability and completeness of the proposed methods of data collection to ensure they are feasible for a definitive trial
(6) To gather data to allow estimation of the sample size requirements for the definitive trial
(7) To determine the practicalities of delivering the required number of HENRY programmes within the trial period in regards to programme implementation

### Eligibility criteria

#### Local authorities

To be eligible, local authorities had to allow randomisation of four/six children centres and be willing to have their staff trained to deliver HENRY or use external teams outside of the centres. Local authorities had to be HENRY ‘naive’, meaning they had never delivered HENRY or trained staff to deliver HENRY prior to the study, or contain clusters of children’s centres that were naïve to HENRY. For the purposes of this study, HENRY naïve clusters were defined as a group of centres that did not include any centres that were either (a) delivering HENRY at the time of the study, or within the past 2 years, or (b) had been trained to deliver HENRY within the past 2 years. Local authorities that were not situated in an area with coverage of NatCen were not eligible.

#### Children’s centres

Eligible centres were children’s centre or early years setting, ready to start the HENRY programmes within 4 weeks of training completion, each capable of delivering three programmes throughout the study. Centre managers had to agree to support participant recruitment in their centre. Children’s centres were excluded from the study if they had delivered HENRY programmes, or their staff had attended HENRY training in the last 2 years.

#### Parents

Eligible parents or carers were those with a preschool child (aged 6 months to 5 years), willing to attend the programme (in HENRY centres), provide data for the study and be English speaking or agree to take an interpreter to the intervention and data collection. Parents were excluded if they had severe learning difficulties, their child was tube fed or had known clinical conditions likely to affect growth or had attended the HENRY programme for a previous child. In HENRY centres, only parents who enrolled to attend a HENRY programme were eligible to take part. No restriction on attendance at any other programmes was enforced in either treatment allocation.

### Recruitment, participants and setting

Seven local authorities that were planning to commission HENRY during the study period were invited to take part in the study initially via conversations with HENRY national office. In addition, 33 ‘Expressions of interest’ forms were sent out to all local authorities across England and Wales. Children’s centres within each local authority were nominated by commissioning leads within each area. Once childcare centres had agreed to participate, parent recruitment took place via invitations from staff and through poster advertisements. Centre staff asked potential participants to provide consent for their contact details to be shared with field researchers from NatCen (http://natcen.ac.uk/) (a social research unit working across the UK), who then made contact to arrange a visit at the parents’ home or children’s centre. Eligibility was confirmed and participants consented to take part in the study during these visits prior to collection of data. Eligible and consenting participants were registered by an authorised NatCen researcher using the CTRU automated 24-h registration system (Gen 24). Participants received a £10 shopping voucher per visit.

### Randomisation

To reduce contamination, children’s centres were randomised as clusters. Six centres in each local authority were randomised to the intervention or control in a 1:1 allocation ratio (HENRY; control) following baseline data collection using minimisation incorporating a random element. This ensured that treatment groups were balanced for size of children’s centre (number of permanent centre members of staff not including staff using the centre such as health visitors and nursery workers) (≤ eight / > eight members of staff); area level ethnicity (< 80% / ≥ 80% White British (census data)); and area level deprivation (≤ 10% / > 10% ranking within Index of Multiple Deprivation at the Lower Super Output Area). Randomisation was carried out by the statistician (MC) at the Clinical Trials Research Unit (CTRU) at the University of Leeds. Local authorities and children’s centres were notified of treatment allocation directly from researchers at the CTRU. Staff at six children’s centres allocated to the active treatment arm were trained to deliver the HENRY intervention and staff at the other six children’s centres received no HENRY training and acted as control centres.

### Blinding

It was not possible to blind children’s centre staff and participants to treatment allocation, but data collection researchers were blinded to treatment allocation. By design, they only became unblinded during the final follow up interview, when requesting consent to share HENRY attendance data. The feasibility of recording and dealing with unblinding incidents was assessed, in which y NatCen staff were asked to report unblinding and if needed, assign a different follow up interviewer.

### Intervention

Parents in the intervention arm attended the 8-week HENRY (Health, Exercise, Nutrition for the Really Young) programme delivered in children centre’s to groups of 8–10 parents. Programme details are documented elsewhere [13, 18, 19]. In brief, it aims to provide parents with the skills and knowledge to support a healthy lifestyle in preschool children and their families. Training for intervention delivery is split into two stages: (1) *Centre level training*: equipping staff with skills and knowledge to promote and provide healthy nutrition in early years settings and to support parents to provide healthy lifestyle and nutrition for their families; and (2) *Practitioner level training to deliver HENRY programme to families*: training staff to deliver the 8 week programme. Both types of training are underpinned by a combination of proven models of behaviour change, including the Family Partnership Model, motivational interviewing and solution focused support. Topics covered in the HENRY programme include eating habits, balancing healthy meals and snacks, child appropriate portion sizes, emotional wellbeing, parenting skills and activity. Services provided by children’s centres that were deemed similar to those of HENRY (e.g. parenting, healthy eating), as well as services attended by study participants, were recorded.

### Control group

Standard care was continued in centres assigned to the control condition. These centres delivered all their usual programmes (including programmes such as ‘stay and play’, ‘cook and eat’, baby massage and other parenting courses). Staff did not receive HENRY training or materials. Following all data collection, centres in the control group received free HENRY training to enable delivery of the HENRY sessions. Parents in control centres were offered attendance at HENRY once the data collection for the study had ended (i.e. a waiting list). All services similar to those of the HENRY programme that were provided by the centres were recorded (both in the control centres and those allocated to HENRY).

### Data collection and outcomes

NatCen researchers were responsible for collecting data (usually in participants homes) and were required to attend training prior to baseline and follow up data collection, where they completed a practical assessment of data collection procedures for height, weight and waist circumference. Additionally, random observations of data collection were conducted by research field leads to ensure protocols were adhered to. Measurements and questionnaires were completed at baseline (within a 6 week window prior to each HENRY programme, over 5 periods of programme delivery) and follow up at 11 months. Following data collection, researchers sent completed data collection booklets to the NatCen head office for data checking and completed booklets were then sent to CTRU via a secure transfer encrypted system using a data specification document provided by CTRU. The CRFs contained no identifiable information (unique identifiers only). Unscheduled forms, such as participant withdrawal or researcher un-blinding, were mailed directly from the NatCen interviewers to the CTRU as the event occurred. HENRY data were transferred to CTRU.

### Primary objectives

#### Objective 1

Recruitment rate of local authorities, children’s centres and parents were assessed through analysis of process data and data routinely collected data from HENRY central office, including number of local authorities/parents screened for eligibility, reasons for ineligibility and numbers (and reason for) declining.

### Progression rules for definitive trial

In order to progress to the definitive phase III trial, the following criteria were applied:

Green (fully feasible):

- Recruitment of two local authorities within 12 months
- Randomisation of at least 12 children’s centres within 12 months
- An average of at least 4 parents registered per programme (or control group equivalent)

Amber (modifications required):

- Randomisation of 8–12 children’s centres within 12 months
- Three parents registered per programme

Red (not feasible):

- Recruitment of less than two local authorities (and their service providers (if applicable)) within 12 months
- Randomisation of less than 8 children’s centres within 12 months
- Less than 3 parents registered per programme

#### Objective 2

Training and quality assurance indicators were reported by NatCen head office and collected from HENRY process data, including length of time taken to train and certify staff and the time lapse between centre randomisation and HENRY programme delivery.

#### Objective 3

Local authority preferences for commissioning HENRY within the context of a trial.

We explored the appetite of local authority involvement in a future definitive trial via two methods. Direct conversations were conducted informally between local authority commissioners and members of the HENRY national office team as part of their on-going commissioning process over the course of the feasibility study. In addition, we sent out expression of interest surveys electronically to 150 directors of children’s services in local authorities across the country in February 2020 (with a reminder two weeks later). This survey (14/15-items) provided an overview of the study requirements and invited commissioner to provide information to establish trial eligibility of the local authority prior to requesting a statement of interest. Given the transient nature of roles within local authorities, we were unable to confirm whether surveys reached our intended people of people within a commissioning role.

#### Objective4

Contamination identification and risk, ascertained using a mixed methods design. Quantitative data determined the number of parents registered to control centres who attended centres running HENRY, the extent to which HENRY trained facilitators worked in or visited control centres and the types of other programmes delivered in centres that focused on lifestyle change. Qualitative interviews sought to find evidence of sharing of HENRY messages by staff and/or parents in HENRY centres to staff and/or parents allocated to control centres.

### Secondary objectives

#### Objective 5

Feasibility of methods and data collection was assessed from qualitative and quantitative data. Qualitative interviews were undertaken with local authorities, children’s centre managers, children’s centre staff and study participants who were asked to provide feedback on the acceptability of study methods and measures. Quantitative data included number, proportion and timing of parent withdrawals from HENRY programme (intervention centres only), follow-up data collection (parents registered to HENRY and control centres), reasons for withdrawal and the amount of missing data.

#### Objective 6

Determination of sample size for a definitive trial was estimated using feasibility data of gender adjusted body mass index (BMI) in both arms, difference between arms and 95% confidence intervals, estimation of clustering effect (ICC) and cluster size.

#### Objective 7

Intervention compliance and implementation (attendance and fidelity) was estimated from routinely gathered data on the timing of delivery of first HENRY programme (plus reasons if delayed); number of HENRY courses delivered per centre; attendance rates at HENRY programmes and reasons for absence; and routinely gathered implementation checklists and audit data used by HENRY national office to monitor fidelity of programme delivery.

### Outcome measures intended for the definitive trial

The following data were collected to determine their feasibility for inclusion in a future definitive trial:

Intended primary outcome: Reference child BMI z-score (age and gender adjusted height(m)/weight(kg)^2^) measured by NatCen interviewers.

Intended secondary outcomes:

- Primary caregiver BMI (measured height(m)/weight(kg)^2^) and waist circumference (cm)
- Family eating/activities, via the validated Golan Family Eating and Activity Habits Questionnaire [20]
- Parenting self-efficacy, via the Dumka Parenting Self Agency Measure [21]
- Feeding, via the Baughcum pre-schooler feeding questionnaire [22]
- Dental health via a bespoke questionnaire based on the Dental Health Survey of Children and Young People developed by the School of Dentistry at the University of Leeds, o measure the potential wider impact of HENRY on a child’s dental health (Dental Questionnaire)
- Centre policy and practices via a bespoke environment questionnaire
- Quality of life (EQ-5D) [23]
- Health care resource use data (via a bespoke questionnaire) for the child and the parent within the NHS (health services, hospital, social services) as well as time-off work in relation with HENRY

### Process evaluation

A process evaluation aimed to assess the acceptability of the research methods, the fidelity of HENRY delivery and the extent (and impact) of contamination. We followed the Medical Research Council guidance (2015) and measured relevant constructs proposed by Baranowski and Stables [24]. This evaluation also facilitated accurate reporting requirements of the TiDieR checklist [23]. Qualitative data for the process evaluation were collected by CTRU researchers.

### Sample size

The planned sample size for the feasibility study was 120 parents across 2 local authorities. These numbers provide sufficient confidence that a phase III multi-site trial could be successfully conducted [25, 26] and meet the recommendation that at least 60 participants per group are required when estimating study summary measures [27]. A formal power calculation was not appropriate as effectiveness is not being evaluated.

### Analyses

Quantitative analyses for objectives 1, 2, 3 (primary objectives) and 5, 6 7 (secondary objectives): focussed on descriptive statistics and confidence interval estimation. Continuous outcomes (such as time to delivery) were summarised using the mean, standard deviation, median and interquartile range. Categorical outcomes (such as reasons unwilling to provide consent) were summarised using the number and percentage of responses for each category. For objectives 5 and 6, the number of parents or children with missing outcome data was reported for each outcome. Outcomes were presented overall, by arm and where relevant by children’s centre (cluster) for baseline and follow-up time points, using the intention-to-treat population. No formal hypothesis testing was conducted as part of this feasibility study.

Analysis of the qualitative data (objectives 4, 5 and 7) was conducted in NVivo data analysis software [28] and was guided by a deductive organising framework devised from the research objectives (contamination and acceptability) and interview and focus group topic guides. Inductive thematic analysis was then applied to identify codes and sub-codes, and potential relationships between these codes [29]. Qualitative data were coded by two members of the research team, who conducted an independent check of 10% of the transcripts. Analysis was discussed in team meetings and consensus was reached for the content of themes and their influence on the subsequent design of a definitive trial.

## Results

### Participant flow and feasibility of recruitment (objective 1)

**Fig. 1:**
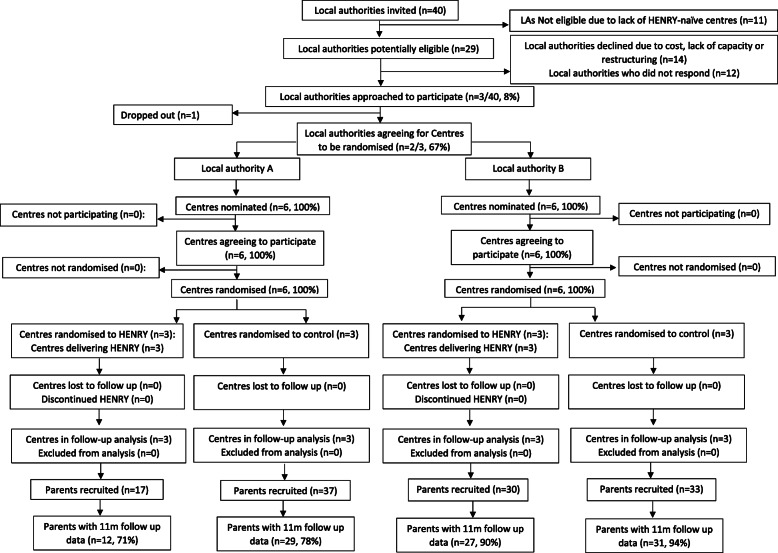
Summary of participant flow

Figure 1 provides a summary of participant flow. Three local authority areas (7.5%) expressed an interest in taking part (one via HENRY commissioning and two via EOI letters); one area subsequently declined participation and two agreed to take part. Of the remaining areas, 11 were ineligible (29.7%), 14 declined participation due to cost/lack of capacity/restructuring (37.8%) and 12 did not respond (32.4%). No further local authorities were approached after the required number were recruited (*n* = 2).

Recruitment of local authorities and centres took place between August 2017 and April 2018 (time from initial contact until signing all 26 contracts). The two recruited local authorities contributed 12 children’s centres (six per local authority), all of which were randomised in December 2017 and April 2018 respectively.

**Fig. 2:**
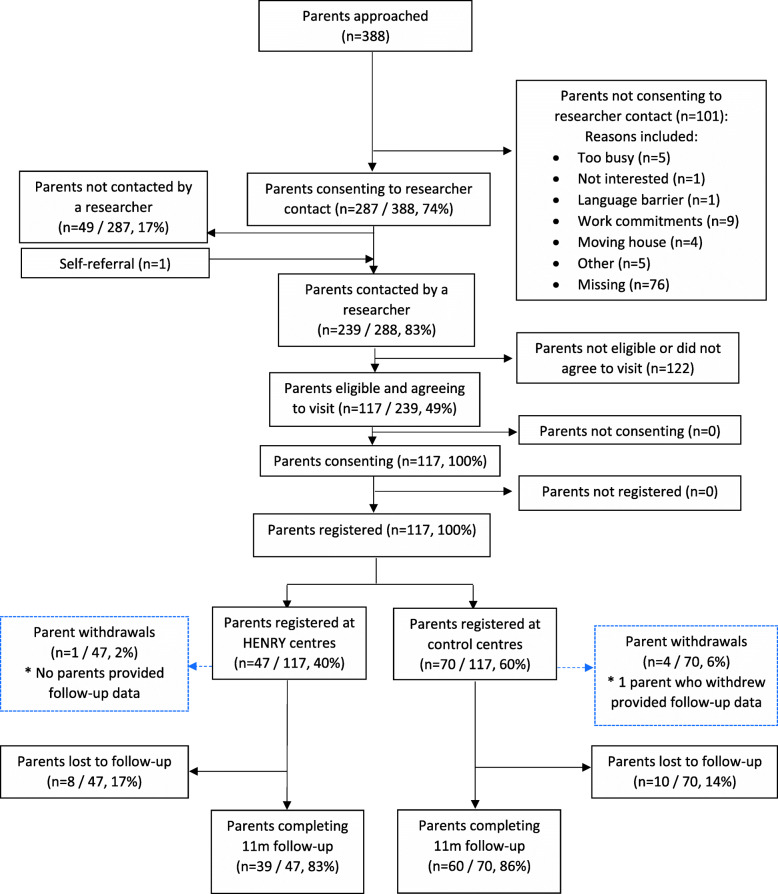
Parental/carer screening and study flow

Parent screening and recruitment took place between March and November 2018 in one local authority and between May and September 2018 in another. Recruitment of parents coincided with planned HENRY programmes (i.e. within a 6 week window prior to each programme or equivalent). During the feasibility study, one site ran two programmes and the other ran three programmes; providing a total of five periods of 6-week recruitment cycles. Across all five periods, 388 parents were approached for entry into the study (Fig. 2) and provided some level of screening data. A total of 287 parents (74.0% of screened) were willing to be contacted by a researcher to discuss the study in more detail. For the 101 parents unwilling to provide consent to researcher contact, reasons were listed as ‘work commitments’ (8.9%), ‘too busy’ (5.0%), ‘moving house’ (4.0%), ‘not interested’ (1.0%), ‘language barrier’ (1.0%) and ‘other’ (5.0%). Reasons were missing for 75.2% of those who did not provide consent to researcher contact. Field researchers contacted 239 parents (83.0% of those willing to be contacted plus one additional parent who self-referred), of whom, 117 were deemed to be eligible (29.9% of approached, 40.4% of those who consented to contact, plus one additional parent who self-referred). All 117 parents consented to take part and were registered to the study between June 2018 and November 2018. The study recruited 117 of the planned 120 parents across 30 programmes (or control group equivalents) resulting in an overall average of 3.9 parents registered per programme. Data were collected by 11 NatCen researchers.

In terms of our a priori progression criteria, this study met the green criteria for recruitment of local authorities (two local authorities (and their service providers (if applicable)) within 12 months). However, parent recruitment equated to an average of 3.9 parents per programme which met the amber criteria (three parents registered per programme).

### Baseline data

Recruited local authorities were based in two distinct areas in northern and central England. Centres were located in areas of high deprivation, with all but one in areas with an index of multiple deprivation (IMD) score of one (IMD scores range from 1 to 9 with 1 being the most deprived and 9 being the least). Parent characteristics are shown in Table 1. The majority of participants were female (97%), aged 25–34 years (68%) and 63% identified themselves as being White British. Children were mostly aged 2 years or younger (84%) with approximately similar numbers of boys and girls. Child ethnicity (not shown) closely resembled that of the parent/caregiver. Around 60% of children had siblings, who also lived in the same house.

**Table 1:** Baseline demographic characteristics

	HENRY	Control	Total
Parent/caregiver age			
18–24 years	6 (12.8%)	9 (12.9%)	15 (12.8%)
25–34 years	34 (72.3%)	45 (64.3%)	79 (67.5%)
35–44 years old	6 (12.8%)	16 (22.9%)	22 (18.8%)
45–54 years old	1 (2.1%)	0 (0.0%)	1 (0.9%)
55+ years	0 (0.0%)	0 (0.0%)	0 (0.0%)
Total	47 (100%)	70 (100%)	117 (100%)
Parent/caregiver gender			
Male	0 (0.0%)	4 (5.7%)	4 (3.4%)
Female	47 (100.0%)	66 (94.3%)	113 (96.6%)
Total	47 (100%)	70 (100%)	117 (100%)
How did participant learn about the study?			
Poster/leaflet in Children’s Centre	7 (13.7%)	19 (26.4%)	26 (21.1%)
Introduction at the beginning of group session	3 (5.9%)	19 (26.4%)	22 (17.9%)
Social media	0 (0.0%)	0 (0.0%)	0 (0.0%)
Children's Centre website	7 (13.7%)	4 (5.6%)	11 (8.9%)
Member of staff outside of a group session	19 (37.3%)	20 (27.8%)	39 (31.7%)
Other	15 (29.4%)	10 (13.9%)	25 (20.3%)
Total	51 (100%)	72 (100%)	123 (100%)
Relationship to child			
Mother	45 (95.7%)	66 (94.3%)	111 (94.9%)
Father	0 (0.0%)	4 (5.7%)	4 (3.4%)
Step–mother/father	0 (0.0%)	0 (0.0%)	0 (0.0%)
Other	1 (2.1%)	0 (0.0%)	1 (0.9%)
Missing	1 (2.1%)	0 (0.0%)	1 (0.9%)
Total	47 (100%)	70 (100%)	117 (100%)
Ethnicity			
English/Welsh/Scottish/Northern Irish/British	27 (57.4%)	47 (67.1%)	74 (63.2%)
Indian	3 (6.4%)	4 (5.7%)	7 (6.0%)
Pakistani	7 (14.9%)	5 (7.1%)	12 (10.3%)
Bangladeshi	1 (2.1%)	1 (1.4%)	2 (1.7%)
Chinese	1 (2.1%)	1 (1.4%)	2 (1.7%)
African	0 (0.0%)	3 (4.3%)	3 (2.6%)
Caribbean	2 (4.3%)	3 (4.3%)	5 (4.3%)
Mixed ethnicity/Multiple other	0 (0.0%)	1 (1.4%)	1 (0.9%)
White other	5 (10.6%)	5 (7.1%)	10 (8.5%)
Other	1 (2.1%)	0 (0.0%)	1 (0.9%)
Total	47 (100%)	70 (100%)	117 (100%)
Child age			
6–11 months	19 (40.4%)	19 (27.1%)	38 (33.0%)
12–23 months	12 (25.5%)	24 (34.3%)	36 (31.3%)
2 years	5 (10.6%)	19 (27.1%)	24 (20.9%)
3 years	7 (14.9%)	4 (5.7%)	11 (9.6%)
4 years	2 (4.3%)	2 (2.9%)	4 (3.5%)
Missing^a^	2 (4.3%)	2 (2.9%)	4 (3.5%)
Total	47 (100%)	70 (100%)	117 (100%)
Child gender			
Male	20 (42.6%)	36 (51.4%)	56 (47.9%)
Female	24 (51.1%)	34 (48.6%)	58 (49.6%)
Missing	3 (6.4%)	0 (0.0%)	3 (2.6%)
Total	47 (100%)	70 (100%)	117 (100%)
Other children in house			
Yes	30 (63.8%)	38 (54.3%)	68 (58.1%)
No	17 (36.2%)	32 (45.7%)	49 (41.9%)
Missing	0 (0.0%)	0 (0.0%)	0 (0.0%)
Total	47 (100%)	70 (100%)	117 (100%)

^a^For 2 children (1 child in the HENRY arm and 1 child in the control arm), age was collected but due to data inaccuracies, it was set to missing

### Training and quality assurance (objective 2)

Sixty-five members of staff attended the core HENRY training across both local authorities, of whom, 21 attended training to deliver the HENRY parent programme and were certified as facilitators. The time from the point of signing contracts to deliver HENRY (including time to train and certify staff) ranged from 1 to 39 weeks (median 27 weeks). This extended period was reported to be due to a lag between the delivery of core training and group facilitator training in some centres.

### Local authority preferences for commissioning HENRY (objective 3)

Over the course of the feasibility study, representatives from nine eligible local authorities expressed an interest in being involved in a potential future definitive trial of HENRY. The majority of these were expressed via our web-based survey, in which 43 (29%) local authority representatives who were sent the survey reviewed the study information, 16 (11%) completed items up to the point of eligibility and seven (5%) provided data to indicate eligibility and were interested in participating in a future evaluation. These local authorities reported managing a range of between six and 38 children centres each. Two other local authorities expressed an interest following routine commissioning conversations with HENRY central office.

### Sources and risk of contamination (objective 4)

Quantitative and qualitative data both indicated that contamination was probable within this setting; however, parents were unable to confirm whether or not this had an impact on their behaviours. This has been described elsewhere in detail [Stamp et al., submitted at same time to Pilot and Feasibility Studies]. In brief, there was potential for HENRY messages to be shared through communications between parents, sharing of knowledge from centre staff, staff working between multiple centres and staff working between control and intervention centres during the study period. There was a high degree of political and structural change within early year settings during the study period due mainly to austerity. As a result of this, two of the study centres merged together, one of which had been allocated to HENRY and one allocated to control. Consequently, three parents (4%) who were registered within a control centre attended the HENRY programme. Interviews revealed that 11 parents (16%) who were registered to control centres had friends who had attended the HENRY programme and 17 (24%) said they had friends who attend other children’s centres. Additionally, 34 (72%) parents attending HENRY and 54 (77%) attending control centres reported that they attended other programmes during the study. Parenting programmes (*n* = 21), baby sensory sessions (*n* = 18) and diet and lifestyle programmes (*n* = 15) were most commonly attended. Staff appeared to present the highest risk of contamination, predominantly due to working between control and intervention centres. Parents did share HENRY messages; however, these appeared to be within their friends and family network in response to people asking questions as opposed to voluntarily sharing messages to a wider audience (e.g., social media). Thus, risk of contamination of HENRY messages from the parents in the intervention group to control group parents was lower due to the randomisation at the children centre level.

### Feasibility of data collection and methods (objective 5)

#### Quantitative data

Baseline questionnaires were received for 116 participants (99%) and only the self-efficacy questionnaire was missing for the remaining participant. BMI z-score could not be derived for 32 (27%) children, mostly due to field researchers being unable to record child height. Follow-up data were collected from 99 parents (85%); 39 (83%) parents registered to HENRY centres and 60 (86%) parents registered to control centres; thus, loss-to-follow-up was 15%. Seven questionnaires were expected for each participant at follow-up; 690 (87%) of 791 expected were received (excluding questionnaires not due as a result of withdrawal). We were unable to calculate BMI z-score at follow-up for 26 children (22% of registered). Five (4%) parents withdrew from the study. Reasons for withdrawal included ‘parent moving into a refuge’, ‘didn’t want child going to nursery to allow attendance at HENRY programme’, ‘no reason given’, ‘newborn and family. None of the children’s centres withdrew their participation.

#### Qualitative data

Two local authority commissioner interviews were conducted in June 2018, 12 weeks after randomisation of children’s centres (to allow time for centre set-up). In addition, staff (*n* = 13) and manager (*n* = 6) interviews took place between April 2019 and September 2019. Two parent focus groups took place in October 2018 (*n* = 4), one in each local authority. Parent interviews (*n* = 16) took place between April 2019 and September 2019. The research methods were deemed acceptable.

##### Commissioners

Commissioners viewed participation in the feasibility study as a positive opportunity for their local authority.

In terms of the study itself, well we thought it was a really good, first and foremost, a really good chance to get involved in a study, which is always good, but we thought it would also help shape come of our practices (local authority commissioner)

One expressed that more clarity around staff roles and responsibilities would have been useful.

I think there’s so much information to take on and understanding what people’s roles were, I think it would be better maybe to have a couple of hand on briefing sessions before the study (local authority commissioner)

##### Staff and managers

A number of staff reported that they felt confident in their understanding of the study and what was expected of them; however, some staff members were less confident with paper work completion during parent recruitment and the research terminology used in RCT’s (i.e. the concept of the control arm and an intervention arm). Having a clear point of contact for such queries enhanced confidence of the children’s centre staff role.

It was a little bit tricky, I think only because I hadn’t done a feasibility study before and I know that members of the team maybe felt a bit tricky and just sort of remembering which were the control sites and what a control site meant and what a delivery site meant… (Staff member)

Staff felt confident in recruiting parents and identified the most common barrier for parent recruitment was English not being a first language, or difficulty understanding the study due to low literacy levels.

We have to explain to the parents what this about. Cos you know the parents here, English is their second language (Staff member)

In these scenarios, they suggested using alternatives such as a video participant information sheet to compliment the paper documents. Staff also reported that parents raised concerns about sharing their personal information.

##### Parents

The majority of parents told us that data collection appointments were easy to organise and they appreciated having the option for these to be at home or at their local children’s centre. Parents also reported that they were happy with the measurements and the questions that they were asked, though a small number commented on the high number of questions.

It was fine yeah the woman was really friendly, talked us through everything, and put us at ease about the questions and doing the weight measurements and stuff like that (Parent).

Oh it was very long, lots of questions […] it was a very lengthy booklet that they went through. Some of the questions, I think that it was a bit too much (Parent).

The HENRY intervention received positive feedback from most parents.

I really liked the portion sizes, you know when we were going through the food and stuff like that, I found that really, really helpful, we started doing something on the back of that as well was just rewarding each other (Parent)

They showed me how to make a meal times more better for children, how much portions they should eat, don’t give them too less, don’t give them too much and how to balance it […]. They have given me a lot of recipes to try. That’s a lot of things that helped me (Parent).

### Sample size determination for definitive trial (objective 6)

Although the feasibility study was not powered to provide a precise estimate of the level of clustering relating to group effects, the results provide an indication of the level of clustering by children’s centre, average cluster size and the variability of the planned primary outcome of BMI z-score. The mean BMI z-score was 0.6 at baseline (1.8 HENRY vs 0.0 control) and 0.6 at follow-up (0.9 HENRY vs 0.5 control). The standard deviation for the baseline BMI z-score was 2.34 (2.47 HENRY vs. 2.05 control) based on available data (*n* = 85/117). At follow-up, the standard deviation for the BMI z-score was 1.45 (1.75 HENRY vs. 1.22 control; *n* = 91/117). The intra cluster correlation coefficient (ICC) of the adjusted BMI (z-score) was estimated to be less than 0.01 for the children’s centres. The average cluster size (number of parents recruited per centre over 2–3 programmes) was 9.8 overall; 7.8 in HENRY centres and 11.7 in control centres. Cluster size ranged between 4 and 25 parents overall; between 4 and 12 parents in HENRY centres and between 4 and 25 in control centres. The number of children included in the calculation of the ICC for adjusted BMI in each cluster ranged from 3 to 19, with an average of 7.6 children in each cluster.

Using a two-sided 5% significance level, assuming 20% loss to follow-up and adjusting for clustering effects, a phase III trial would require a target sample size of 1248 (624 per arm) to detect a standardised effect size of 0.3 with 90% power. The adjustment for clustering assumes an ICC of 0.05, a coefficient of variation of 0.48 and an average cluster size of 24 parents recruited in each of the 52 Children’s Centres.

### Intervention compliance and implementation (participant attendance and fidelity) (objective 7)

#### Compliance

Only one of the six HENRY centres delivered their first programme within 4 weeks of completing training. The remaining centres delivered their first programme 6–7 weeks after training. A total of 14 HENRY programmes were delivered during the study.

#### Attendance

As noted above, we requested separate consent to access linked attendance data at follow-up. Only 15 (32%) parents consented to have their attendance data shared; these were spread across centres and local authorities and were broadly similar in characteristics to those who did not consent. It was only possible to link attendance data for 14 parents. Among these, parents attended between five and eight HENRY sessions (average 6.3 sessions). We also obtained anonymous group level data from all the sessions delivered in centres during the study which included parents who were not participating in the study. These data indicate that an average of 4.9 parents attended each HENRY programme (range of 2 to 10 parents) with an average of 3.6 parents completing the 8-week programme (attending at least 5 out of 8 sessions) (range of 1 to 8 parents). Reasons were not often provided for non-attendance although family issues and illness/change of job were reported.

#### Fidelity

Routinely gathered implementation checklists and audit data monitored by HENRY central office indicated that all programmes had been delivered with fidelity.

### Trends in outcome measures intended for the definitive trial

**Fig. 3:**
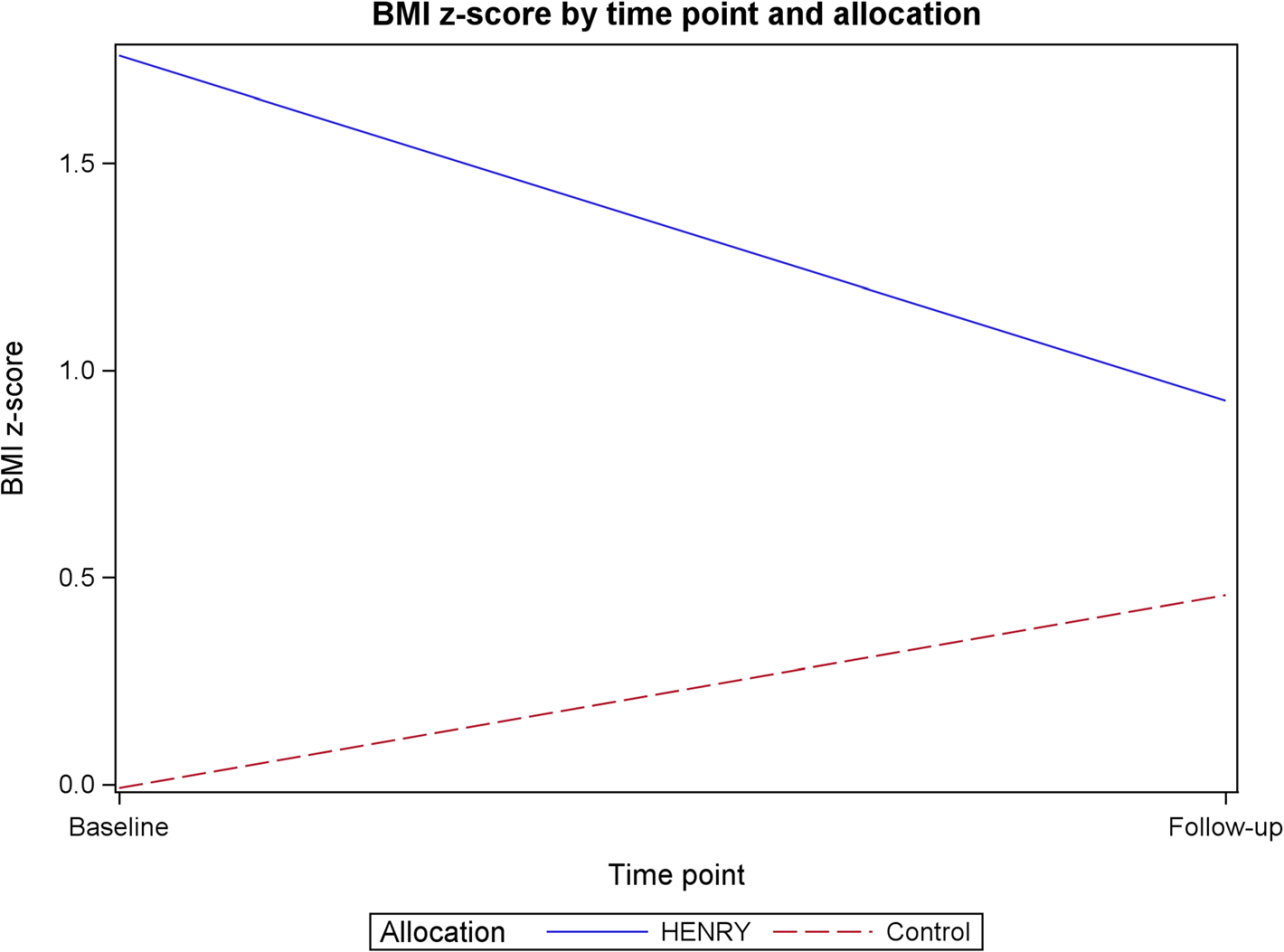
Mean BMI z-score at baseline and follow-up by allocation

Data collected from intended outcome measures are provided in Tables 2 and 3 (and supplementary Table 1), including the amount of missing data. The intended primary outcome data for a definitive trial is BMI z-score. At baseline, children within HENRY centres had a higher average BMI z-score than those in control centres (1.8 (SD 2.47) vs. − 0.0 (SD 2.05), indicating a selection bias. At follow-up, BMI z-score in children within HENRY centres children reduced to 0.9 (SD 1.75) and increased to 0.5 (SD 1.22) in control centres (Fig. 3).

**Table 2:** Parent and child physical measures

	Baseline			Follow up		
	HENRY	Control	Total	HENRY	Control	Total
Child height						
Mean (s.d.)	83.6 (12.42)	85.7 (10.23)	85.0 (10.95)	90.2 (10.88)	91.7 (8.90)	91.1 (9.69)
Median (range)	83.0 (57.8, 07.0)	86.3 (67.0, 13.5)	85.9 (57.8, 13.5)	89.3 (71.0, 15.0)	91.0 (77.6, 19.8)	89.8 (71.0, 119.8)
Missing	19^a^	10	29^a^	9	11	20
*N*	28	60	88	38	59	97
Child weight						
Mean (s.d.)	12.2 (4.01)	11.7 (2.88)	11.9 (3.37)	15.0 (4.06)	14.3 (2.65)	14.6 (3.26)
Median (range)	11.9 (6.5, 28.1)	11.7 (6.3, 19.9)	11.8 (6.3, 28.1)	14.7 (8.8, 24.4)	14.0 (9.5, 21.2)	14.0 (8.8, 24.4)
Missing	3	4	7	9	10	19
*N*	44	66	110	38	60	98
Child BMI						
Mean (s.d.)	19.1 (4.28)	16.4 (2.51)	17.3 (3.41)	18.4 (4.34)	17.0 (1.79)	17.6 (3.11)
Median (range)	17.9 (14.2, 33.2)	16.6 (7.6, 21.4)	17.1 (7.6, 33.2)	17.3 (11.6, 37.9)	16.9 (12.8, 21.3)	17.1 (11.6, 37.9)
Missing	19	12	31	9	11	20
*N*	28	58	86	38	59	97
Child BMI z-score						
Mean (s.d.)	1.8 (2.47)	− 0.0 (2.05)	0.6 (2.34)	0.9 (1.75)	0.5 (1.22)	0.6 (1.45)
Median (range)	0.9 (− 2.0, 9.6)	0.4 (− 8.7, 2.9)	0.6 (− 8.7, 9.6)	0.9 (− 3.8, 5.9)	0.6 (− 3.4, 2.8)	0.6 (− 3.8, 5.9)
Missing	19	13	32	13	13	26
*N*	28	57	85	34	57	91
Parent/carer weight^a^						
Mean (s.d.)	76.2 (23.11)	76.3 (18.48)	76.3 (20.31)	80.3 (26.41)	78.1 (20.17)	79.0 (22.80)
Median (range)	70.2 (39.8, 135.2)	72.3 (47.9, 125.7)	72.3 (39.8, 135.2)	74.5 (41.6, 133.9)	75.9 (50.5, 124.1)	74.8 (41.6, 133.9)
Missing	3	1	4	8	13	21
*N*	44	69	113	39	57	96
Parent/carer waist circumference^b^						
Mean (s.d.)	97.5 (19.96)	99.6 (22.88)	98.8 (21.72)	95.1 (27.21)	95.3 (16.29)	95.2 (21.30)
Median (range)	94.0 (68.0, 155.0)	97.0 (68.0, 177.0)	95.0 (68.0, 177.0)	91.9 (43.0, 161.6)	95.0 (53.2, 146.2)	92.8 (43.0, 161.6)
Missing	7	7	14	11	18	29
*N*	40	63	103	36	52	88
Parent/carer height^a^						
Mean (s.d.)	161.5 (6.84)	163.5 (9.75)	162.7 (8.73)	–	–	–
Median (range)	161.5 (148.0, 174.0)	164.0 (113.0, 195.4)	163.0 (113.0, 195.4)	–	–	–
Missing	3	3	6	–	–	–
*N*	44	67	111	–	–	–
Parent/carer BMI						
Mean (s.d.)	29.2 (8.04)	28.4 (6.81)	28.7 (7.29)	30.9 (9.46)	28.5 (6.56)	29.5 (7.90)
Median (range)	27.4 (15.7, 49.1)	26.9 (19.0, 58.0)	27.1 (15.7, 58.0)	29.9 (16.5, 49.9)	27.0 (20.3, 46.7)	27.2 (16.5, 49.9)
Missing	4	3	7	10	16	26
*N*	43	67	110	37	54	91

^a^Data was set to missing for 2 children for child height at baseline (2 in the HENRY arm), 1 child (in the control arm) for child weight at baseline, 2 parents for parent height at baseline (2 in the control arm), and 1 parent for parent weight at baseline (in the control arm). Overall, parent and child demographics were similar for those where child BMI z-score could be calculated and those for whom child BMI-z score was missing

^b^Parent/carer waist circumference was an optional measure for participants

**Table 3:** Participant-reported outcome measures at baseline and follow-up

	Baseline					Follow up		
	HENRY	Control		Total		HENRY	Control	Total
Parent self-efficacy^a^								
Mean (s.d.)	19.9 (2.92)	20.8 (2.52)		20.4 (2.71)		20.2 (3.14)	20.4 (3.06)	20.4 (3.08)
Median (range)	20.0 (13.0, 25.0)	21.0 (15.0, 25.0)		20.0 (13.0, 25.0)		21.0 (12.0, 25.0)	20.5 (14.0, 25.0)	21.0 (12.0, 25.0)
Missing	1	2		3		8	10	18
*N*	46	68		114		39	60	99
FEAQ overall score^b^								
Mean (s.d.)	40.9 (21.93)	43.9 (18.88)		42.7 (20.13)		39.0 (21.02)	42.1 (16.01)	40.8 (18.11)
Median (range)	40.0 (-27.0, 109.0)	44.0 (-8.0, 109.0)		43.0 (-27.0, 109.0)		40.0 (-21.0, 88.0)	43.0 (12.0, 98.0)	41.0 (-21.0, 98.0)
Missing	0	0		0		8	10	18
*N*	47	70		117		39	60	99
Pre-school feeding overall score^c^								
Mean (s.d.)	1.0 (0.51)	1.1 (0.43)		1.0 (0.46)	1.0 (0.48)	1.0 (0.47)	1.0 (0.47)	
Median (range)	1.0 (0.3, 2.2)	1.0 (0.2, 2.0)	1.0 (0.2, 2.2)	1.0 (0.4, 2.1)	1.0 (0.3, 2.4)	1.0 (0.3, 2.4)		
Missing	0	0	0	9	10	19		
*N*	47	70	117	38	60	98		
EQ-5D-3L overall score^d^								
Mean (s.d.)	0.8 (0.20)	0.9 (0.17)	0.9 (0.19)	0.9 (0.17)	0.9 (0.18)	0.9 (0.18)		
Median (range)	0.8 (0.3, 1.0)	1.0 (− 0.0, 1.0)	1.0 (− 0.0, 1.0)	0.8 (0.1, 1.0)	1.0 (− 0.2, 1.0)	1.0 (− 0.2, 1.0)		
Missing	1	0	1	1	4	5		
*N*	46	70	116	38	56	94		

^a^Parent self-efficacy ranges from 5 to 25, with higher scores indicating greater parenting self-efficacy

^b^Family Eating and Activity Habits Questionnaire (FEAQ) overall score shown, with a higher score reflecting less appropriate eating patterns (no maximum score)

^c^Pre-schooler feeding overall score ranges from 0 to 4, with higher scores indicating poorer feeding practices

^d^EQ-5D-3L ranges from -0.594 to 1 with a higher score indicating better quality of life

## Discussion

This study provides important preliminary evidence which indicates that a cluster RCT of HENRY to assess its impact on childhood obesity prevention is feasible. Importantly, this feasibility stage has enabled us to consider methods and timelines to efficiently deliver the definitive trial within a public health setting so that findings are able to inform local and national decision making in the commissioning and delivery of the HENRY programme. Our recruitment data indicated a substantial level of willingness to take part; however, the progression criteria for parent recruitment fell just short of target, placing it within an ‘amber’ range (average of 3.9 parents per programme compared to target of 4 parents per programme). Importantly, the feasibility study provided valuable insight to ensure that targets can be met in the definitive trial. For example, we identified some issues with the time frame that was applied to contacting parents who had expressed an interest to take part, which could be alleviated by extending recruitment windows. Seventy four percent of parents who were approached in the centres agreed to be contacted by a researcher and 49% of those contacted were registered. Recruitment to community based interventions is notoriously challenging, particularly related to those that seek to engage under-served populations [30–32]. Our recruitment rate of 49% is similar or higher than others reported in the literature [33–36] and all participants were recruited from centres based within the highest levels of deprivation in the UK. Seventy four percent of parents who were approached in the centres agreed to be contacted by a researcher and 49% of those contacted were registered. Recruitment to community based interventions is notoriously challenging, particularly related to those that seek to engage under-served populations [30–32]. Our recruitment rate of 49% is similar or higher than others reported in the literature, with other UK based feasibility studies reporting between 30-48% [33–36].

We also found that recruitment in control centres was greater than that in HENRY centres; primarily because of the wider pool of participants available in the control centres (i.e. not restricted to only those parents who had booked to attend the HENRY programme). A future trial may need to investigate methods to cap participant screening in control centres.

It is promising that participant retention was high in this study, with 83% and 86% of follow-up questionnaires received in HENRY and control arms respectively. On the whole, missing data was also very low for questionnaire data. However, greater amount of physical measurement data were missing, including child height data, which was missing for a considerable number of children at baseline. This was discovered early in the study and enabled us to provide additional training prior to follow-up data collection. Data collection using an external research company worked well as field researchers were local to study participants allowing multiple contact attempts to be made. This is the first randomised study to utilise NatCen data collection and the future trial protocol will allow in depth briefings and closer monitoring to ensure that all field researchers adhere to and understand CTRU standards. Our process evaluation found that field staff sometimes conducted visits when children were sleeping, and that additional training needs were identified for measurement of length as well as height. Further, our retrospective method to capture consent for HENRY attendance data was inefficient and a future trial would need to ensure that this is included in the initial consent process.

This feasibility study identified other key areas (not necessarily pre-defined by our research objectives) which need to be considered to optimise the design of the future trial. Of particular relevance, our baseline primary outcome data differed between treatment arms, with control children having a BMI z-score near to zero and HENRY children having a greater BMI z-score. Our follow-up data indicated that children whose parents attended the HENRY programme had a trend for BMI z-score reduction towards an ideal weight (for their age and gender) and excess weight gain (an increase in BMI z-score) in control children. If this selection bias was repeated in a future definitive trial, the primary outcome of ‘difference between BMI z-score at follow-up’ may not show the true effect of the HENRY programme. Similar trends were also observed for parenting self-efficacy data, (consistent with other evidence [37]). Our process data and data from an ethnography of children’s centres [38] indicates that, although HENRY is intended to be offered universally, many areas target enrolment to those parents in greatest need; thus, generating a selection bias. In other words, participants recruited in HENRY centres may be characteristically different to those recruited in the control centres. This bias is an inherent risk in cluster randomised designs when participant recruitment occurs post randomisation [39] and the design of the future RCT needs to minimise this bias using techniques such as withholding randomisation allocation from children’s centres until after parent screening and/or altering parent recruitment practices or amending parent eligibility criteria. Further, BMI z-score has been criticised for lack of robustness in the context of severe obesity [40], although the feasibility study noted only 14.5% children were above the 95^th^ percentile for BMI. BMI z-scores have also been commended for their high interpretability and generalisability [41]. It is however possible that a primary outcome of BMI z-score is not appropriate given a decrease in BMI z-score may be undesirable in underweight children.

The value of including behavioural questionnaires (including feeding practices) is uncertain given the lack of any trends observed within our feasibility study. However, data collection methods were well accepted by participants and further discussion is warranted to determine their inclusion for a fully powered trial. Conversely, through discussions with our Trial Steering Committee (TSC) and other collaborators, a future trial will be designed which includes additional assessment of the potential wider impact of (and consequences on) HENRY. In order to deliver the programme, centre staff must first receive core training in the overall HENRY approach, in which holistic, centre level approaches are advocated (impacting on the physical and social environment of the centre). Feedback from practitioners who have been trained to deliver HENRY indicates that it has the potential to impact on their personal lives [19] and data collected from our research in early years settings [38, 42] (including this feasibility study) clearly highlights the effects that national and local policy can have on the implementation of HENRY. Measurement of these system wide factors will therefore be essential in order to provide context around the findings of the definitive trial.

## Conclusion

Conducting a feasibility study to evaluate the childhood obesity prevention programme ‘HENRY’ has been an invaluable process; not only to ensure its viability moving forward but also to allow us to refine the protocol to enhance the quality of a future definitive trial. In particular, whilst the methods appeared to be feasible and acceptable, consideration needs to be given to reducing selection bias, optimising data collection protocols for primary outcome data, and reducing contamination.

## Supplementary Information

**Additional file 1:.** Table S1. Dental questionnaire (supplementary table)

## Data Availability

Original data is stored in a secure database within the University of Leeds. Scored and cleaned data, as well as output for analyses, are available upon request from the study PI, Maria Bryant
