# Supplementary material for: Cluster randomised controlled feasibility study of HENRY: a community-based intervention aimed at reducing obesity rates in preschool children
Source: Pilot Feasibility Stud. 2021 Feb 26;7:59. doi: 10.1186/s40814-021-00798-z (PMC7908721; doi:10.1186/s40814-021-00798-z)
Supplement: Supplementary file 1 — Additional file 1:. Table S1. Dental questionnaire (supplementary table) [file 40814_2021_798_MOESM1_ESM.docx]

**Table S1. Dental questionnaire (supplementary table)**

|  | Baseline | | | Follow-up | | |
| --- | --- | --- | --- | --- | --- | --- |
|  | HENRY | Control | Total | HENRY | Control | Total |
| Age of child when brushing started |  |  |  |  |  |  |
| Under 6 months | 4 (8.5%) | 18 (25.7%) | 22 (18.8%) | 6 (15.8%) | 13 (21.7%) | 19 (19.4%) |
| Between 6 months to 1 year of age | 34 (72.3%) | 36 (51.4%) | 70 (59.8%) | 23 (60.5%) | 38 (63.3%) | 61 (62.2%) |
| Between 1 and 2 years of age | 5 (10.6%) | 9 (12.9%) | 14 (12.0%) | 7 (18.4%) | 8 (13.3%) | 15 (15.3%) |
| Between 2 and 4 years of age | 0 (0.0%) | 0 (0.0%) | 0 (0.0%) | 1 (2.6%) | 1 (1.7%) | 2 (2.0%) |
| 4 years of age or older | 0 (0.0%) | 0 (0.0%) | 0 (0.0%) | 0 (0.0%) | 0 (0.0%) | 0 (0.0%) |
| My child does not brush their teeth or have them brushed for them | 3 (6.4%) | 6 (8.6%) | 9 (7.7%) | 1 (2.6%) | 0 (0.0%) | 1 (1.0%) |
| Missing | 1 (2.1%) | 1 (1.4%) | 2 (1.7%) | 0 (0.0%) | 0 (0.0%) | 0 (0.0%) |
| Total | 47 (100%) | 70 (100%) | 117 (100%) | 38 (100%) | 60 (100%) | 98 (100%) |
| Who brushes child's teeth? |  |  |  |  |  |  |
| Your child | 18 (38.3%) | 8 (11.4%) | 26 (22.2%) | 15 (39.5%) | 14 (23.3%) | 29 (29.6%) |
| An adult | 8 (17.0%) | 23 (32.9%) | 31 (26.5%) | 3 (7.9%) | 14 (23.3%) | 17 (17.3%) |
| An adult and your child together | 16 (34.0%) | 27 (38.6%) | 43 (36.8%) | 19 (50.0%) | 32 (53.3%) | 51 (52.0%) |
| Not applicable | 1 (2.1%) | 4 (5.7%) | 5 (4.3%) | 0 (0.0%) | 0 (0.0%) | 0 (0.0%) |
| Missing | 4 (8.5%) | 8 (11.4%) | 12 (10.3%) | 1 (2.6%) | 0 (0.0%) | 1 (1.0%) |
| Total | 47 (100%) | 70 (100%) | 117 (100%) | 38 (100%) | 60 (100%) | 98 (100%) |
| How often are teeth brushed? |  |  |  |  |  |  |
| More than three times a day | 1 (2.1%) | 2 (2.9%) | 3 (2.6%) | 1 (2.6%) | 1 (1.7%) | 2 (2.0%) |
| Three times a day | 4 (8.5%) | 1 (1.4%) | 5 (4.3%) | 0 (0.0%) | 2 (3.3%) | 2 (2.0%) |
| Twice a day | 17 (36.2%) | 34 (48.6%) | 51 (43.6%) | 23 (60.5%) | 43 (71.7%) | 66 (67.3%) |
| Once a day | 18 (38.3%) | 21 (30.0%) | 39 (33.3%) | 13 (34.2%) | 13 (21.7%) | 26 (26.5%) |
| Less than once a day | 2 (4.3%) | 1 (1.4%) | 3 (2.6%) | 0 (0.0%) | 1 (1.7%) | 1 (1.0%) |
| Not Applicable | 1 (2.1%) | 4 (5.7%) | 5 (4.3%) | 0 (0.0%) | 0 (0.0%) | 0 (0.0%) |
| Missing | 4 (8.5%) | 7 (10.0%) | 11 (9.4%) | 1 (2.6%) | 0 (0.0%) | 1 (1.0%) |
| Total | 47 (100%) | 70 (100%) | 117 (100%) | 38 (100%) | 60 (100%) | 98 (100%) |
| Does your child visit the dentist? |  |  |  |  |  |  |
| Yes, for check-ups | 23 (48.9%) | 35 (50.0%) | 58 (49.6%) | 31 (81.6%) | 42 (70.0%) | 73 (74.5%) |
| Yes, only when they have trouble with their teeth | 0 (0.0%) | 1 (1.4%) | 1 (0.9%) | 1 (2.6%) | 2 (3.3%) | 3 (3.1%) |
| No, they have never been | 23 (48.9%) | 33 (47.1%) | 56 (47.9%) | 6 (15.8%) | 16 (26.7%) | 22 (22.4%) |
| Missing | 1 (2.1%) | 1 (1.4%) | 2 (1.7%) | 0 (0.0%) | 0 (0.0%) | 0 (0.0%) |
| Total | 47 (100%) | 70 (100%) | 117 (100%) | 38 (100%) | 60 (100%) | 98 (100%) |
| Has child had anaesthetic for dental work? |  |  |  |  |  |  |
| Yes | 6 (12.8%) | 6 (8.6%) | 12 (10.3%) | 3 (7.9%) | 5 (8.3%) | 8 (8.2%) |
| No | 33 (70.2%) | 49 (70.0%) | 82 (70.1%) | 30 (78.9%) | 44 (73.3%) | 74 (75.5%) |
| N/A | 8 (17.0%) | 14 (20.0%) | 22 (18.8%) | 5 (13.2%) | 10 (16.7%) | 15 (15.3%) |
| Missing | 0 (0.0%) | 1 (1.4%) | 1 (0.9%) | 0 (0.0%) | 1 (1.7%) | 1 (1.0%) |
| Total | 47 (100%) | 70 (100%) | 117 (100%) | 38 (100%) | 60 (100%) | 98 (100%) |
